# A Systematic Review of the Effect of Gene–Lifestyle Interactions on Metabolic-Disease-Related Traits in South Asian Populations

**DOI:** 10.1093/nutrit/nuae115

**Published:** 2024-09-16

**Authors:** Manahil M Bineid, Eduard F Ventura, Aryan Samidoust, Venkatesan Radha, Ranjit Mohan Anjana, Vasudevan Sudha, Gemma E Walton, Viswanathan Mohan, Karani Santhanakrishnan Vimaleswaran

**Affiliations:** Hugh Sinclair Unit of Human Nutrition, Department of Food and Nutritional Sciences, Institute for Cardiovascular and Metabolic Research (ICMR), University of Reading, Reading RG6 6DZ, United Kingdom; Department of Biotechnology, Institute of Agrochemistry and Food Technology-National Research Council (IATA-CSIC), 46980 Valencia, Spain; Hugh Sinclair Unit of Human Nutrition, Department of Food and Nutritional Sciences, Institute for Cardiovascular and Metabolic Research (ICMR), University of Reading, Reading RG6 6DZ, United Kingdom; Department of Molecular Genetics, Madras Diabetes Research Foundation, ICMR Centre for Advanced Research on Diabetes, Chennai 603103, India; Department of Molecular Genetics, Madras Diabetes Research Foundation, ICMR Centre for Advanced Research on Diabetes, Chennai 603103, India; Department of Foods, Nutrition and Dietetics Research, Madras Diabetes Research Foundation, Chennai 600086, India; Department of Diabetology, Dr Mohan’s Diabetes Specialties Centre, IDF Centre of Excellence in Diabetes Care, Chennai 600086, India; Department of Foods, Nutrition and Dietetics Research, Madras Diabetes Research Foundation, Chennai 600086, India; Food Microbial Sciences Unit, Department of Food and Nutritional Sciences, University of Reading, Whiteknights, Reading RG6 6AP, United Kingdom; Department of Molecular Genetics, Madras Diabetes Research Foundation, ICMR Centre for Advanced Research on Diabetes, Chennai 603103, India; Department of Foods, Nutrition and Dietetics Research, Madras Diabetes Research Foundation, Chennai 600086, India; Department of Diabetology, Dr Mohan’s Diabetes Specialties Centre, IDF Centre of Excellence in Diabetes Care, Chennai 600086, India; Hugh Sinclair Unit of Human Nutrition, Department of Food and Nutritional Sciences, Institute for Cardiovascular and Metabolic Research (ICMR), University of Reading, Reading RG6 6DZ, United Kingdom; The Institute for Food, Nutrition and Health (IFNH), University of Reading, Reading RG6 6AH, United Kingdom

**Keywords:** nutrigenetics, systematic review, South Asia, gene–lifestyle interaction, metabolic disease, dietary intake, physical activity

## Abstract

**Context:**

Recent data from the South Asian subregion have raised concern about the dramatic increase in the prevalence of metabolic diseases, which are influenced by genetic and lifestyle factors.

**Objective:**

The aim of this systematic review was to summarize the contemporary evidence for the effect of gene–lifestyle interactions on metabolic outcomes in this population.

**Data sources:**

PubMed, Web of Science, and SCOPUS databases were searched up until March 2023 for observational and intervention studies investigating the interaction between genetic variants and lifestyle factors such as diet and physical activity on obesity and type 2 diabetes traits.

**Data extraction:**

Of the 14 783 publications extracted, 15 were deemed eligible for inclusion in this study. Data extraction was carried out independently by 3 investigators. The quality of the included studies was assessed using the Appraisal Tool for Cross-Sectional Studies (AXIS), the Risk Of Bias In Non-randomized Studies—of Interventions (ROBINS-I), and the methodological quality score for nutrigenetics studies.

**Data analysis:**

Using a narrative synthesis approach, the findings were presented in textual and tabular format. Together, studies from India (n = 8), Pakistan (n = 3), Sri Lanka (n = 1), and the South Asian diaspora in Singapore and Canada (n = 3) reported 543 gene–lifestyle interactions, of which 132 (∼24%) were statistically significant. These results were related to the effects of the interaction of genetic factors with physical inactivity, poor sleep habits, smoking, and dietary intake of carbohydrates, protein, and fat on the risk of metabolic disease in this population.

**Conclusions:**

The findings of this systematic review provide evidence of gene–lifestyle interactions impacting metabolic traits within the South Asian population. However, the lack of replication and correction for multiple testing and the small sample size of the included studies may limit the conclusiveness of the evidence. Note, this paper is part of the Nutrition Reviews Special Collection on Precision Nutrition.

**Systematic Review Registration:**

PROSPERO registration No. CRD42023402408.

## INTRODUCTION

The prevalence of metabolic diseases, including obesity and type 2 diabetes (T2D), has reached epidemic proportions globally, notably in the South Asian (SA) population, who are known to have an increased propensity for diabetes.^1–5^ Data compiled between 2009 and 2019 from the SA subregion, comprising India, Pakistan, Bangladesh, Sri Lanka, Bhutan, Maldives, Nepal, and Afghanistan, have raised concerns about the spiked increase in attributable deaths due to diabetes in these countries.^6^^,^^7^ In particular, India accounts for the highest mortality due to T2D,^8^ which can be traced to unique anthropometric, biochemical, and clinical characteristics, including a higher degree of insulin resistance^5^ and increased abdominal fat and central obesity rates.^9^^,^^10^ Additionally, early impairment of beta cell function was recorded among SA individuals, even with mild dysglycemia.^11^ This unique profile has been described as the “Asian Indian Phenotype,” making the population more susceptible to diabetes even at a younger age and with a lower body mass index (BMI) compared with other ethnic populations.^12–15^ Nevertheless, the rising prevalence of the disorder was recorded in individuals of SA origin both in their native communities and in the diaspora when compared with the general population.^15–19^ This impacts global health due to the significant number of SA migrants living in Western countries.^1^

The rising diabetes and obesity burden in SA results from urbanization and rapid lifestyle changes, characterized by a shift toward Westernized dietary patterns, sedentary lifestyles, and physical inactivity.^1^^,^^20^^,^^21^ Moreover, the SA diet is typically higher in carbohydrates, trans fats, and saturated fats, and has a lower daily consumption of fruits, vegetables, and dietary fiber compared with other populations.^18^^,^^22^ Additionally, studies have reported increased refined sugar and processed food intakes during recent decades.^23^ However, it has been suggested that multifactorial metabolic conditions originate to a great extent from a complex interaction between genetic and lifestyle factors.^24–29^ Although research findings have shown that lifestyle modifications can effectively reduce the incidence of diabetes,^30^^,^^31^ the response to lifestyle interventions differs between individuals and specific ethnic groups due to the influence of genetic variations.^32^^,^^33^

To this end, nutrigenetic studies have revealed the distinct impact of lifestyle factors on the genetic susceptibility of various populations to metabolic diseases.^34^^,^^35^ Thus, the discovery of gene–lifestyle (G–L) interactions provides a comprehensive understanding of the pathogenic mechanisms underlying obesity and diabetes phenotypes; however, it remains a challenging task to explain the basis for these interactions and identify which gene and genetic variants are involved in the interaction with lifestyle factors.^29^^,^^36^ Understanding the drivers of increased metabolic risk in high-risk populations and identifying G–L interactions may help optimize prognostic tools and enable targeted intervention approaches that are particularly effective in a population subgroup of similar genetic makeup.^16^^,^^34^^,^^37^ Furthermore, data from nutrigenetic studies can be fed into systems that employ modern technology, such as artificial intelligence and machine learning, to predict disease risk and mitigate the burden of noncommunicable diseases.^38^ Although some systematic reviews on G–L interactions have been conducted in other populations^36^^,^^39–42^ only one mini-review has focused on the SA population.^34^ Thus, to fill the gap in the literature, the aim was to systematically review studies investigating the effects of interactions of genetic variants with diet, physical activity (PA), and other lifestyle factors on metabolic traits, including obesity and T2D in populations from the southern subregion of Asia.

## MATERIALS AND METHODS

This systematic review was conducted following PRISMA guidelines^43^ and synthesis without meta-analysis (SWiM) in systematic reviews reporting guidelines.^44^ The PICOS (population, intervention, comparison, outcomes, and study design) criteria used for study selection are outlined in Table 1. The protocol for this systematic review was previously registered in the International Prospective Register for Systematic Review (PROSPERO, registration number CRD42023402408).

**Table 1.** PICOS Criteria for the Inclusion of Studies

Parameter	Inclusion criterion
Population	SA population, including India, Bangladesh, Pakistan, Sri Lanka, Bhutan, Maldives, Nepal, Afghanistan and the South Asian diaspora
Intervention or exposure	Lifestyle factors such as dietary factors, PA, smoking and sleep patterns, and genetic factors
Comparison	Interactions between genetic susceptibility and exposure to lifestyle factors
Outcomes	T2D and obesity-related traits
Study design	Observational and intervention studies published in English

*Abbreviations:* SA, South Asia; PA, physical activity; T2D, type 2 diabetes.

### Eligibility criteria

Articles investigating the interaction between genetic variations and lifestyle factors, including diet and PA, on metabolic traits in SA populations from Bangladesh, Pakistan, Sri Lanka, Bhutan, Maldives, India, Nepal, and Afghanistan or SA diaspora were eligible for inclusion. The articles were in English and included observational and intervention studies. Studies that did not investigate the effects of G–L interactions on T2D or obesity or did not include SA populations were excluded.

### Search strategy

Until March 2023, literature searches were conducted independently by M.M.B, E.F.V, and A.S. in MEDLINE (via PubMed), Web of Science, and SCOPUS until literature saturation. At that point, the included publications had also been searched for potential articles in reference lists. The search strings were created using Medical Subject Headings (MeSH) vocabulary and other terms, adapted to follow the Peer Review of Electronic Search Strategies (PRESS) guideline,^45^ which can be found in Table S1.

### Study selection

**Figure 1.**
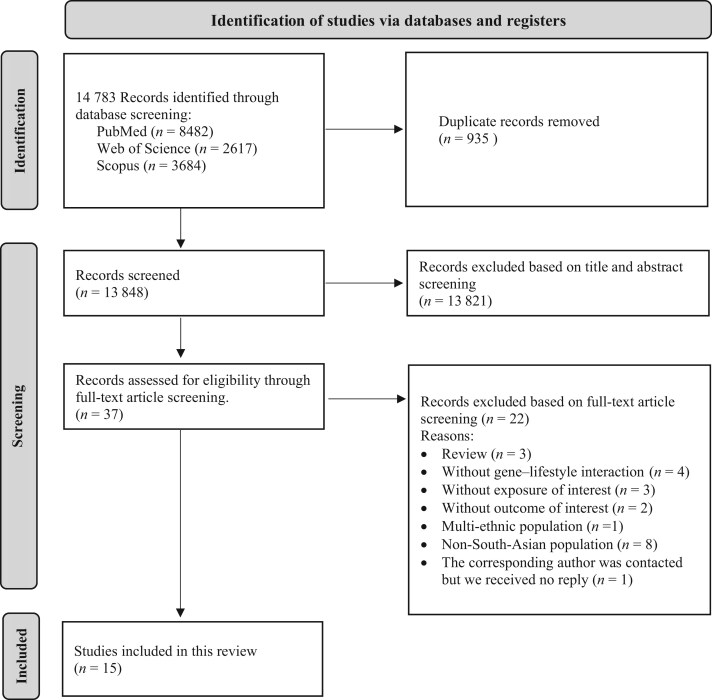
PRISMA Flow Diagram of Identification and Selection of Studies and Reasons for Excluding Studies. The literature search was conducted in MEDLINE (via PubMed), Web of Science, and SCOPUS search engines up until 15 March 2023

Duplicate records were removed using EndNote software.^46^ Considering the pre-established inclusion criteria shown in Table 1, titles and abstracts were blindly screened by the 3 reviewers (M.M.B, E.F.V, and A.S.), followed by full-text screening and discussion until reviewers reached a consensus. The authors were contacted if the information required to determine the study’s eligibility was unavailable. Reasons for study exclusion were (1) without G–L interaction; (2) without exposure/outcome of interest; (3) non-South Asian population; and (4) not original articles (Figure 1). Additionally, one paper was excluded due to missing data, as there was no response from the corresponding author.^47^

### Data extraction

The 3 reviewers (M.M.B, E.F.V, and A.S.) extracted data from the included literature with Microsoft Excel 2023 software.^48^ A narrative synthesis of the findings was performed following the SWiM in the Systematic Reviews guideline.^44^ The reviewers ensured consistency in the extracted data; this included population characteristics, lifestyle factors, study designs, genetic variations, metabolic traits, and *P*-values for the effects of G–L interactions on metabolic traits (Table 2). *P*_interaction_ refers to the *P*-values for effects of G–L interactions on metabolic markers; *P*-values < .05 were considered statistically significant. In addition, a graphical representation of the significant interactions was visualized through a heat map, where color intensity represented the *P*-value for the effects of the interaction, and the darker the color, the lower the *P*-value. All heat maps were created using the ggplot2 package in R software^49^ within an RStudio environment.^50^ Meta-analysis was not possible, given that the included studies investigated several dietary factors, genetic variants, and metabolic traits, in addition to methodological heterogeneity.

**Table 2.** Characteristics of Individual Studies

Country/cohort name	Study design	Population/sample size (age)	SNPs or GRS	Exposure/assessment tool	Outcomes	Replication	Reference
India/Chennai Urban Rural Epidemiology Study (CURES)	C-S	Indian/*n* = 548 (222 NGT, 152 pre-diabetic, 176 T2D) (≥20 y)	*FTO*-GRS; *FTO* rs8050136 and rs2388405	Dietary (fat, protein, and carbohydrates energy intake %)/interviewer-administered validated FFQ PAL/self-reported validated questionnaire	Obesity (WC, WHR, BMI), diabetes (FG, FI, HbA1C)	No	Surendran et al (2019a)^57^
		Indian/*n* = 545 (219 NGT, 151 pre-diabetic, 175 T2D) (≥20 y)	5-SNP Metabolic-GRS; *FTO* rs8050136 and rs2388405; *TCF7L2* rs12255372 and rs7903146 and *MC4R* rs17782313	Dietary (fat, protein, and carbohydrates energy intake%)/interviewer-administered validated FFQ PAL/self-reported validated questionnaire	Obesity (WC, BMI), diabetes (FG, FI, HbA1C)	No	Alathari et al (2020)^58^
		Indian/*n* = 1062 (406 NGT, 566 T2D) (45 ± 12 y)	7-SNP GRS; *TCF7L2* rs12255372 and rs7903146; *FTO* rs8050136, rs918031, rs1588413, rs7193144 and rs1076023 3-SNP GRS; *TCF7L2* rs12255372 and rs7903146; *FTO* rs8050136	Dietary (fat, protein, and carbohydrates energy intake %, animal protein [g] and plant protein [g])/interviewer-administered validated FFQ	Obesity (WC, BMI), diabetes (FG, FI, HbA1C)	No	Alsulami et al (2021)^59^
		Indian/*n* = 1682 (821 NGT, 861 T2D) (>20 y)	*MC4R* rs17782313, *TCF7L2* rs12255372, and rs7903146	Dietary (fat, protein, carbohydrates, fiber, PUFA, and MUFA intake [g])/interviewer-administered validated FFQ PAL/self-reported validated questionnaire	Obesity (WC, BMI), diabetes (FG, FI, HbA1C, T2D risk)	No	Bodhini et al (2017)^56^
		Indian/*n* = 497 (237 NGT, 260 T2D) (44 ± 10 y)	3-SNP GRS; *CETP rs4783961*; and *LPL* rs327, and rs3200218	Dietary (fat, protein, carbohydrates, fiber, SFA, MUFA, PUFA [g])/interviewer-administered validated FFQ PAL/self-reported validated questionnaire	Obesity (WC, WHR, BMI, common obesity)	No	Wuni et al (2022)^61^
		Indian/*n* = 1618 (884 NGT, 734 T2D) (≥20 y)	*FTO* rs8050136 and rs11076023	Dietary (fat, protein, and carbohydrates energy intake (%), fiber (g), energy-adjusted glycemic index, energy-adjusted glycemic load)/interviewer-administered validated FFQ PAL/self-reported validated questionnaire	Obesity (WC, BMI, obesity risk), diabetes (FG, FI, HbA1C, T2D risk)	No	Vimaleswaran et al (2016)^25^
India/Chennai Urban Rural Epidemiology Study (CURES)	C-S	Indian/*n* = 1886 (945 NGT, 941 T2D) (≥20 y)	*Omentin A326T* rs2274911	Dietary (fat, protein, and carbohydrates energy intake (%), fiber (g)/interviewer-administered validated FFQ PAL/self-reported validated questionnaire	Obesity (WC, WHR, BMI), diabetes (FG, FI, fasting serum adiponectin, HbA1C)	No	Vimaleswaran et al (2021)^60^
India	C-C	Indian/*n* = 1379 (758 T2D patients, 621 normal controls) (≥40 y)	15 SNPs; *TCF7L2* rs7903146, rs11196205, rs12255372, *IGF2BP2* rs4402960, rs1470579, *SLC30A8* rs13266634, *CDKAL1* rs7754480, rs7756992, *CDKN2A*/B rs10811661, *HHEX* rs1111875, rs7923837, IRS*-*1 rs1801278, *CAPN10* rs3792267, rs5030952, *PPARG* rs1801282	Dietary (food habits, junk food, alcohol consumption)/questionnaire Lifestyle (PA, smoking, migration pattern)/questionnaire	Diabetes (T2D risk)	No	Uma Jyothi and Reddy, (2015)^55^
Sri Lanka/Genetic of Obesity and Diabetes (GOOD)	C-S	Sinhalese/*n* = 109 healthy (25–50 y)	Metabolic-GRS: *FTO* rs9939609 and rs8050136; *MC4R* rs17782313 and rs2229616; *TCF7L2* rs12255372 and rs7903146; *KCNJ11* rs5219; *CAPN10* rs3792267, rs2975760, and rs5030952 B12-GRS: *MTHFR* rs1801133; *CPS1* rs1047891; *CUBN* rs1801222; *CD320* rs2336573; *TCN2* rs1131603; *CLYBL* rs41281112; *FUT2* rs602662; *TCN1* rs34324219; *FUT6* rs778805 and *MUT* rs1141321	Dietary (fat, protein, and carbohydrates energy intake %), fiber (g)/interviewer-administered validated FFQ. PAL/global physical activity questionnaire (GPAQ)	Obesity (WC, WHR, HC, BMI, BF %), diabetes (FG, FI, HbA1C)	No	Surendran et al (2019b)^68^
Pakistan	C-C	Pakistani/*n* = 578 (290 obese cases, 288 control) (≥20 y)	*MC4R* rs17782313, *BDNF* rs6265, *FTO* rs1421085, *TMEM18* rs7561317, *NEGR1* rs2815752	Lifestyle factors (eating pattern, TFDF, diet consciousness, sleep duration, sleep–wake cycle, shift work, low PA)/self-reported questionnaire	Obesity (BF%, BMI, WC, HC, WHR, and WHtR)	No	Rana et al (2021)^64^
Pakistan/Pakistan Risk of Myocardial Infarction Study (PROMIS)	C-C	Pakistani/*n* = 16 157 (7925 MI patients, 8232 controls) (53.8 ± 9.6 y)	95 SNPs GRS based on 95 SNPs	PAL, smoking/validated questionnaire	Obesity (BMI)	No	Ahmad et al (2015)^62^
	C-S	Pakistani/*n* = 14 131 (53.8 ± 9.6 y)	*FLJ33534* rs140133294	PAL, smoking/validated questionnaire	Obesity (BMI)	No	Ahmad et al (2016)^63^
Canada/Toronto Nutrigenomics and Health (TNH) Study	C-S	SA diaspora/*n* = 1639 (174 SA) (20–29 y)	*FTO* rs1558902	Dietary (protein energy intake %)/the Toronto-modified Willett FFQ	Obesity (BMI, WC)	No	Merritt et al (2018)^65^
Singapore/Singapore National Health Survey	C-S	Indian diaspora/*n* = 4107 (598 Indian) (18–69 y)	*PLIN* 11482G > A and 14995A > T	Dietary (fat, SFA, PUFA, MUFA and carbohydrates energy intake %)/validated FFQ	Diabetes (FG, FI, HOMA-IR)	No	Corella et al (2006)^66^
	C-S	Indian diaspora/*n* = 4602 (494 = Asian Indian) (40 ± 12 y)	APOA2 m-*265T* > C rs5082	Dietary (SFA energy intake %)/validated FFQ	Obesity (BMI), diabetes (HOMA-IR)	Yes	Corella et al (2011)^67^

*Abbreviations:* APOA2, apolipoprotein A2; BDNF, Brain-Derived Neurotrophic Factor; BF%, body fat percentage; BMI, body mass index (kg/m^2^); CAPN, Calpain; CDKAL1, CDK5 regulatory subunit-associated protein 1-like 1; CDKN2A, Cyclin Dependent Kinase Inhibitor 2A; *CETP*, cholesteryl ester transfer protein; *CLYBL*, Citramalyl-CoA Lyase; *CPS1*, Carbamoyl-Phosphate Synthase 1; *CUBN*, Cubilin; FFQ, food frequency questionnaire; FG, fasting glucose; FI, fasting insulin; FLJ33534, Putative Uncharacterized Protein FLJ33534; *FTO*, alpha-ketoglutarate dependent dioxygenase; *FUT2*, Fucosyltransferase 2; GRS, genetic risk score; HC, hip circumference (cm); HHEX, Haematopoietically Expressed Homeobox; IGF2BP2, Insulin-Like Growth Factor 2 MRNA Binding Protein 2; IRS-1, insulin receptor substrate 1; *KCNJ11*, Potassium Inwardly Rectifying Channel Subfamily J Member 11; *LPL*, lipoprotein lipase; *MC4R*, melanocortin-4-receptor; *MTHFR*, methylenetetrahydrofolate reductase; MUFA, monounsaturated fatty acid; *MUT*, Methylmalonyl-CoA mutase; *NEGR1*, Neuronal Growth Regulator 1; NGT, normal glucose-tolerant individuals; obesity, obesity-related traits; diabetes, diabetes-related traits; PAL, physical activity level (high, medium, low/sedentary); PLIN1, Perilipin 1; PPARG, Peroxisome Proliferator Activated Receptor Gamma; PUFA, polyunsaturated fatty acid; SA, South Asian; SFA, saturated fatty acid; T2D, type 2 diabetes patients; *TCF7L2*, transcription factor 7-like 2; *TCN2*, transcobalamin 2; TFDF, tendency toward fat-dense food.; *TMEM18*, Transmembrane Protein 18; WC, waist circumference (cm); WHR, waist–hip ratio; WHtR, waist–height ratio; MI, Myocardial Infarction; FUT6, Fucosyltransferase 6; TCN1, Transcobalamin 1; C-S, cross-sectional; GPAQ, Global physical activity questionnaire; HbA1C, Hemoglobin A1c PA, physical activity HOMA-IR, Homeostatic Model Assessment of Insulin Resistance; SNP, single nucleotide polymorphism.

### Risk of bias and quality assessment

The risk of bias (ROB) in the included cross-sectional studies was evaluated using the Appraisal tool for Cross-Sectional Studies (AXIS).^51^ The AXIS appraisal tool is a 20-point questionnaire that assesses the quality and reporting of a study, covering areas such as the Introduction, Methods, Results, Discussion, and other relevant sections. Each question was scored following the scoring system: “yes”=1, and “no” or “do not know”=0. Studies scoring >15 points were classified as high quality, those scoring 10–15 were considered as medium quality, and those scoring <10 points were considered as low quality. The Risk of Bias in Non-randomised Studies-of Interventions (ROBINS-I) tool was also used to assess cohort studies, case–control studies, and nonrandomized trials.^52^^,^^53^ This tool consists of signaling questions that cover 7 domains: confounding, selection of study participants, classification of exposure, deviation from intended exposure, missing data, measurement of outcomes, and selection of the reported result. The assessment options for each signaling question were Yes, Probably Yes, Probably No, No, or No Information. Following a domain-level assessment, the overall ROB was classified into low, moderate, serious, and critical risk (Table S2). The methodological quality of the included studies was further assessed using a scoring system previously employed in genetic association studies.^40^^,^^41^^,^^54^ This score included 8 parameters: interaction as the primary aim of the study, interaction test, correction for multiple testing, correction for ethnicity, Hardy–Weinberg equilibrium, test for group similarity at baseline, sample size, and sufficient methodological details. For each parameter, numeric values were assigned to the categorical responses; “yes”=1, “not known”=0, and “no”=−1. In line with the previous reviews,^40^^,^^41^ the tool’s grading scale was from −8 to 8, and the studies were classified as high quality (6 to 8 points), intermediate quality (2 to 5 points), or poor quality (−8 to 1 point) (Table S3).

## RESULTS

### Characteristics of the included studies

A total of 14 783 records were identified from 3 databases (Figure 1). After removing duplicates, 13 848 articles were screened for title and abstract, and 37 full-text articles were assessed for eligibility. Of those, 15 met the inclusion criteria and were considered in this review. Of the included studies, 8 were in the Indian population,^25^^,^^55–61^ 3 were in the Pakistani population,^62–64^ 3 were in the SA diaspora living in Canada^65^ and Singapore,^66^^,^^67^ and 1 was in the Sinhalese population.^68^ The study design of these articles included 3 case–control studies (20%) and 12 cross-sectional studies (80%). Descriptions of individual studies are shown in Table 2. Dietary protein intake was the most widely investigated dietary factor explored among the studies, followed by carbohydrates, fat, dietary fiber, and alcohol intake. In addition to PA, several lifestyle exposures relating to eating patterns, sleep patterns, shift work, and smoking were studied in the articles. Across the 15 included studies, 135 genetic variants were investigated individually or as a genetic risk score (GRS), with *FTO* and *TCF7L2* single nucleotide polymorphisms (SNPs) being the most studied genetic variations. The GRS is a commonly used approach to assess the combined impact of several genetic factors, each with modest effects. Six included studies employed the GRS approach by adding the number of disease-associated alleles across each SNP.^57–59^^,^^61^^,^^62^^,^^68^ The studies used a relatively similar methodology to calculate the GRS. A value of zero, 1, or 2 was assigned to each SNP, indicating the number of risk alleles present. Subsequently, these values were aggregated by adding the number of risk alleles across all targeted SNPs for each participant. This cumulative score represented the individual’s genetic predisposition to the outcome of interest. Based on the median number of risk alleles, the scores were divided into categories, allowing for the identification of individuals with higher or lower genetic risk for the outcome of interest. Additionally, all studies employed an unweighted method to construct the GRS, primarily due to limited information regarding the effect size of the investigated SNPs within the targeted population.

Concerning the outcome measure, investigated obesity traits included obesity risk, BMI, waist circumference (WC), hip circumference (HC), waist-to-hip ratio (WHR), waist-to-height ratio (WHtR), and percentage body fat (BF%), while T2D-related measured outcomes were mainly fasting glucose (FG), fasting insulin (FI), and HbA1C. The sample size of the studies varied between 109 and 16 157 adults, including both men and women. Regarding the assessment of lifestyle exposures, all studies that measured dietary exposure used an interviewer-administered food frequency questionnaire (FFQ).^25^^,^^56–61^^,^^66^^,^^67^ In a similar scenario, studies investigating PA exposure used self- or investigator-administered questionnaires (Table 2).^25^^,^^55–68^

Additionally, by exploring the genetic model tests chosen for the SNP genotype data, most of the studies (*n* = 10) utilized an additive genetic model (comparison between the 3 genotypes), 3 used a dominant model (comparing individuals with common homozygous genotypes with the combined group of rare homozygotes and heterozygotes), and 2 employed a recessive model (comparing individuals with rare homozygotes genotypes with the combined group of common homozygous and heterozygotes) in their analyses. Rationales for the chosen genetic models were provided in a few studies.

### Study quality and risk of bias

The methodological quality evaluation of G–L research identified 5 as high-quality studies^57^^,^^59^^,^^60^^,^^64^^,^^68^ and 10 as medium-quality studies^25^^,^^55^^,^^56^^,^^58^^,^^61–63^^,^^65–67^ (Table S4). The main reasons for the reduction in methodological quality of the studies were a small sample size and failure to report information about corrections for multiple testing. According to the AXIS ROB assessment, all cross-sectional studies were evaluated as having a low ROB (Table S5). The ROBINS-I tool was applied to assess the ROB for the 3 included case–control studies^55^^,^^62^^,^^64^ (Table S6 and Figure S1). One article,^62^ was judged to have a serious ROB due to high bias in the classification of exposure, while the others^55^^,^^64^ had a moderate ROB.

### Effects of gene–lifestyle interaction on obesity

**Figure 2.**
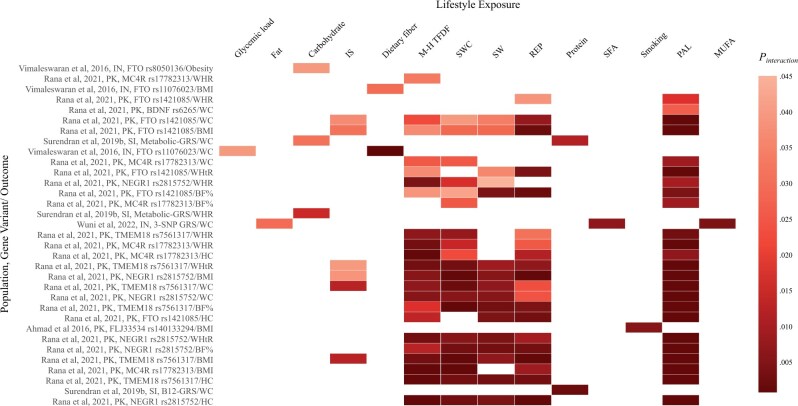
Heat Map Showing the Results of the Significant Interactions Between Gene and Lifestyle Factors in Relation to Obesity in Indian, Sinhalese, and Pakistani Populations. Surendran et al (2029b)^68^: metabolic-GRS=*FTO* (rs9939609, rs8050136); *MC4R* (rs17782313, rs2229616); B12-GRS=*MTHFR* (rs1801133); *CPS1* (rs1047891); *CUBN* (rs1801222); *CD320* (rs2336573); *TCN2* (rs1131603); *CLYBL* (rs41281112); *FUT2* (rs602662); *TCN1* (rs34324219); *FUT6* (rs778805); *MUT* (rs1141321); Wuni et al. (2022)^61^: 3SNP-GRS=*CETP* (*rs4783961*); *LPL* (rs327, rs3200218); Vimaleswaran et al (2016)^25^; Rana et al (2021)^64^; Ahmad et al (2016)^63^. Abbreviations: BF%, body fat percentage; BMI, body mass index; HC, hip circumference; IN, Indian; IS, inadequate sleep; M–H TFDF, a moderate-to-high tendency toward fat-dense food; MUFA, monounsaturated fatty acid; PAL, physical activity level; BDNF, brain-derived neurotrophic factor; FLJ33534, putative uncharacterized protein FLJ33534; FTO, alpha-ketoglutarate dependent dioxygenase; MC4R, melanocortin-4-receptor; NEGR1, Neuronal Growth Regulator 1; TMEM18, Transmembrane protein 18; PK, Pakistani; REP, random eating pattern; SFAs, saturated fatty acids; SI, Sinhalese; SW, shift work; SWC, irregular sleep–wake cycle; WC, waist circumference; WHR, waist–hip ratio; WHtR, waist–height ratio

Of the 15 included studies, 13 investigated the effects of G–L interactions concerning obesity outcomes and reported 120 significant interactions. These findings, including *P*-values for effects of interactions, are shown in Table 3,^25^^,^^63^^,^^64^  Table 4,^61^^,^^68^ and Figure 2.^25^^,^^61^^,^^63^^,^^64^^,^^68^ The results were stratified by lifestyle factors to identify exposure-specific G–L interactions.

**Table 3.** Summary Table of Significant Effects of SNP–Lifestyle Interactions on Obesity Outcomes

Gene and SNP	Reference	Study design	Population (cohort)/sample size	Lifestyle exposure	Obesity outcome	Impact on outcome measures	*P*_interaction_
*FTO* (alpha-ketoglutarate–dependent dioxygenase)							
rs1421085	Rana et al (2021)^64^	C-C	Pakistani/578	Inadequate sleep (<7 h/d)	BMI	↑	.031
					WC	↑	.037
				Low PA	BF%	↑	.004
					WHR	↑	.018
					BMI, HC, WC, WHtR	↑	<.001
				Moderate-to-high TFDF	HC	↑	.013
					WC	↑	.023
					BMI, WHtR	↑	.037
					BF%	↑	.039
				Random eating pattern	BMI, BF%	↑	.002
					HC	↑	.003
					WHtR	↑	.004
					WC	↑	.007
					WHR	↑	.039
				Shift work	BF%, HC	↑	.004
					BMI	↑	.029
					WC	↑	.034
					WHtR	↑	.036
				Irregular sleep–wake cycle	BMI	↑	.029
					WC	↑	.04
					BF%	↑	.042
rs8050136	Vimaleswaran et al (2016)^25^	C-S	Indian (CURES)/1618	Carbohydrate intake % ([T2D; men: 65.50 ± 5.43, women: 64.08 ± 6.27], [NGT; men: 64.60 ± 5.89, women: 63.09 ± 6.84])	Obesity	↑	.04
rs11076023	Vimaleswaran et al (2016)^25^	C-S	Indian (CURES)/1618	Dietary fiber intake (g) ([T2D; men: 28.62 ± 0.54, women: 34.91 ± 0.69], [NGT; men: 29.84 ± 0.42, women: 35.73 ± 0.56])	WC	↓	.0008
					BMI	↓	.03
				Energy-adjusted glycemic load ([T2D; men: 233.52 ± 1.64, women: 226.12 ± 2.40], [NGT; men: 231.63 ± 1.74, women: 224.32 ± 0.36])	WC	ND	.04
*MC4R* (Melanocortin-4-receptor)							
rs17782313	Rana et al (2021)^64^	C-C	Pakistani/578	Low PA	HC	↑	.006
					WC, BF%	↑	.008
					BMI, WHtR	↑	<.001
				Moderate-to-high TFDF	WC	↑	.026
					WHR	↑	.033
					WHtR	↑	.003
					BMI, HC	↑	<.001
				Random eating pattern	BMI	↑	.008
					HC	↑	.011
					WHtR	↑	.026
				Irregular sleep-wake cycle	WHtR	↑	.014
					HC	↑	.023
					WC, BF%	↑	.026
					BMI	↑	<.001
*NEGR1* (Neuronal Growth Regulator 1)							
rs2815752	Rana et al (2021)^64^	C-C	Pakistani/578	Inadequate sleep (<7 h/d)	BMI	↑	.039
				Low PA	WHR	↑	.009
					BF%, BMI, HC, WC, WHtR	↑	<.001
				Moderate-to-high TFDF	WHtR	↑	.003
					WHR	↑	.004
					BMI, WC	↑	.005
					BF%	↑	.012
					HC	↑	.001
				Random eating pattern	BMI, HC	↑	.001
					BF%	↑	.003
					WHtR	↑	.009
					WC	↑	.025
				Shift work	BF%	↑	.003
					HC, WHtR	↑	.004
					BMI, WC	↑	.005
					WHR	↑	.045
				Irregular sleep–wake cycle	BMI	↑	.001
					BF%, HC	↑	.003
					WC, WHtR	↑	.005
					WHR	↑	.017
*TMEM18* (Transmembrane Protein 18)							
rs7561317	Rana et al (2021)^64^	C-C	Pakistani/578	Inadequate sleep (<7 h/d)	BMI, WC	↑	.012
					WHtR	↑	.04
				Low PA	WHR	↑	.003
					BF%, BMI, HC, WC, WHtR	↑	<.001
rs7561317	Rana et al (2021)^64^	C-C	Pakistani/578	Moderate-to-high TFDF	HC	↑	.001
					BMI, WHtR	↑	.003
					WC, WHR	↑	.006
					BF%	↑	.018
				Random eating pattern	BMI	↑	.001
					HC	↑	.003
					BF%	↑	.004
					WHtR	↑	.006
					WC	↑	.024
					WHR	↑	.031
				Shift work	BF%, HC	↑	.003
					BMI, WC	↑	.006
					WHtR	↑	.008
				Irregular sleep–wake cycle	BF%	↑	.001
					WC	↑	.002
					HC, WHtR	↑	.003
					WHR	↑	.007
					BMI	↑	<.001
*FLJ33534* rs140133294	Ahmad et al (2016)^63^	C-S	Pakistani (PROMIS)/14 131	Smoking (ex vs current smokers)	BMI	↓	.0005
*BDNF* rs6265	Rana et al (2021)^64^	C-C	Pakistani/578	Low PA	WC	↑	.027

Outcome measures include obesity traits: ↓ indicates a significant decrease in outcome measure; ↑ indicates a significant increase in outcome measure; ND indicates a non-defined impact. Abbreviations: SNP, single nucleotide polymorphism; BF%, body fat percentage; *BDNF*, brain-derived neurotrophic factor; BMI, body mass index (kg/m^2^); C-C, case–control; C-S, cross-sectional; CURES, Chennai Urban Rural Epidemiology Study; *FLJ33534*, putative uncharacterized protein FLJ33534; HC, hip circumference (cm); NGT, normal glucose tolerance subjects; PA, physical activity; PROMIS, Pakistan Risk of Myocardial Infarction Study; T2DM, type 2 diabetes subjects; TFDF, tendency toward fat-dense food; WC, waist circumference (cm); WHR, waist–hip ratio; WHtR, waist–height ratio.

**Table 4.** Summary Table of Significant Effects of GRS–Lifestyle Interactions on Obesity Outcomes

GRS and SNPs	Reference	Study design	Population (cohort)/sample size	Lifestyle exposure	Obesity outcome	Impact on outcome measures	*P*_interaction_
3-SNP GRS: *CETP rs4783961*; and *LPL* rs327, rs3200218	Wuni et al (2022)^61^	C-S	Indian (CURES)/497	Fat intake (g) (67 ± 27 g)	WC	ND	.03
				MUFA (g) (20 ± 8 g)	WC	ND	.004
				SFA (g) (25 ± 11 g)	WC	↑	.006
B12-GRS: *MTHFR* rs1801133; *CPS1* rs1047891; *CUBN* rs1801222; *CD320* rs2336573; *TCN2* rs1131603; *CLYBL* rs41281112; *FUT2* rs602662; *TCN1* rs34324219; *FUT6* rs778805; *MUT* rs1141321	Surendran, et al (2019 b)^68^	C-S	Sinhalese (GOOD)/109	Protein energy % (11.29 ± 2.31%)	WC	ND	.002
Metabolic-GRS: *FTO* rs9939609 and rs8050136; *MC4R* rs17782313 and rs2229616; *TCF7L2* rs12255372 and rs7903146; *KCNJ11* rs5219; *CAPN10* rs3792267, rs2975760, and rs5030952	Surendran, et al (2019 b)^68^	C-S	Sinhalese (GOOD)/109	Protein energy % (11.29 ± 2.31%)	WC	ND	.011
				Carbohydrate energy % (69.62 ± 8.80%)	WHR	↑	.015
					WC	↑	.031

Outcome measures include obesity traits: ↓ indicates a significant decrease in outcome measure; ↑ indicates a significant increase in outcome measure; ND indicates non-defined impact. Abbreviations: SNP, single nucleotide polymorphism; *CAPN*, calpain; *CETP*, cholesteryl ester transfer protein; *CLYBL*, Citramalyl-CoA Lyase; *CPS1*, Carbamoyl-Phosphate Synthase 1; C-S, cross-sectional; *CUBN*, cubilin; CURES, Chennai Urban Rural Epidemiology Study; *FTO*, alpha-ketoglutarate dependent dioxygenase; *FUT2*, fucosyltransferase 2; GOOD, Genetic of Obesity and Diabetes; GRS, genetic risk score; *KCNJ11*, Potassium Inwardly Rectifying Channel Subfamily J Member 11; *LPL*, lipoprotein lipase; *MC4R*, melanocortin-4-receptor; *MTHFR*, methylenetetrahydrofolate reductase; MUFA, monounsaturated fatty acid; *MUT*, methylmalonyl-CoA mutase; SFA, saturated fatty acid; *TCF7L2*, transcription factor 7-like; *TCN*, transcobalamin; WC, waist circumference (cm); WHR, waist–hip ratio.

#### Effects of interaction between gene and dietary intake on obesity traits

Starting with protein intake, the most commonly investigated dietary factor among the studies, a cross-sectional interaction study of 109 healthy Sinhalese adults from the Genetics of Obesity and Diabetes cohort (GOOD) found that protein–energy intake (%) significantly interacted with a B12-GRS consisting of *MTHFR*, *CPS1*, *CUBN*, *CD320*, *TCN2*, *CLYBL*, *FUT2*, *TCN1*, *FUT6*, and *MUT* variants and a metabolic-GRS based on *FTO*, *MC4R*, *TCF7L2*, *KCNJ11*, and *CAPN10* variants influencing obesity risk.^68^ However, the influence of B12-GRS on WC was evident only under the impact of a high-protein diet. Notably, individuals with ≤9 risk alleles exhibited lower WC when following a high-protein diet in contrast to those consuming a low-protein diet.

Gene–carbohydrate intake interactions were reported in 2 studies.^25^^,^^68^ In the Sinhalese study^68^ an interaction was found between carbohydrate energy intake (%) and the metabolic-GRS on WHR and WC. Among those who consumed a high-carbohydrate diet (78% energy), carriers of ≤8 risk alleles for the metabolic disease had 6.46% lower WHR than individuals with ≥9 risk alleles.^68^ Another cross-sectional study (in 1618 Asian Indians) reported a significant interaction between the *FTO* SNP rs8050136 and carbohydrate energy intake (%) on obesity risk, where individuals carrying the “A” allele had a 2.46-fold higher risk of obesity compared with the “CC” genotype among individuals in the highest tertile of carbohydrate intake (71% energy).^25^ Moreover, interaction of the *FTO* rs11076023 variant with dietary fiber intake (g) was found to decrease obesity, and the “AA” carriers had lower WC and BMI than the “T” allele carriers among those who were in the highest tertile of dietary fiber intake (44 g/d).^25^

Regarding the effect of fat intake, a cross-sectional study using a sample of 497 Asian Indians reported a significant effect of interaction between 3-SNP-GRS (*CETP* and *LPL* variants) and total fat intake (g) on WC; however, this interaction did not remain significant after adjusting for lipid subfractions (high-density lipoprotein, low-density lipoprotein, triglycerides, and total cholesterol).^61^ Additionally, the 3-SNP-GRS interactions with saturated fatty acid (SFA) and monounsaturated fatty acid (MUFA) intake showed a significant effect on obesity, where participants in the low-SFA intake group (≤23.2 g/day), despite carrying ≥2 risk alleles, exhibited a smaller WC in comparison with subjects carrying <2 risk alleles. For those carrying ≥2 risk alleles, high SFA intake (>23.2 g/day) was significantly associated with a larger WC compared to low SFA intake (Table 4). Nevertheless, no further significant gene–diet interaction effects on obesity were found in the SA population.

#### Interactions between genes and physical activity on obesity traits

Significant effects of gene–PA interactions on obesity were reported in 1 case–control study conducted in the Pakistani population with a sample of 578 Pakistani adults^64^ (Table 3). This study established a higher obesity risk that was attributable to the interaction between low PA and 5 genetic variants. The detected interactions between low PA and the *FTO* rs1421085 or the *TMEM18* rs7561317 increased all obesity-related outcomes. Similarly, the risk allele A of the *NEGR1* rs2815752 and the risk allele C of the *MC4R* rs17782313 variants interacted significantly with low PA, resulting in higher BMI, WC, HC, WHR, WHtR, and BF% in this sample. Lastly, an effect of the interaction of *BDNF* SNP rs6265 with PA was observed in increased WC but not in other obesity-related variables.^64^ Another case–control study conducted on a sample of 7925 myocardial infarction cases and 8232 controls recruited from the Pakistani PROMIS cohort revealed effects of several G–L interactions in the control group (mean age 54.1 ± 8.9) between PA and genetic variants, including *CLIP1* rs11057405, *CADM2* rs13078960, and *GALNT10* rs7715256 on BMI.^62^ However, none of these findings remained significant after correction for multiple testing. Further investigations of several genetic variants and GRSs showed no significant effects of interaction with PA in Indian,^25^^,^^57^^,^^58^ Sinhalese,^68^ or Pakistani populations.^63^

#### Effects of interactions between genes and other lifestyle factors on obesity traits

In the case–control study of 578 Pakistani adults, the researchers further investigated the effects of interaction between the 5 SNPs, *MC4R* rs17782313, *FTO* rs1421085, *TMEM18* rs7561317, *NEGR1* rs2815752, and *BDNF* rs6265 and several unhealthy lifestyle factors (including random eating patterns, a moderate-to-high tendency toward fat-dense food, inadequate sleep [<7 hours/day], irregular sleep–wake cycle, and shift work) on obesity risk.^64^ This study identified approximately 84 interactions, as listed in Table 3. The study found that the risk allele “C” of the *MC4R* rs17782313 interacted with random eating patterns, a tendency toward fat-dense food, and irregular sleep, leading to an increase in BMI, HC, and WHtR. Specifically, interactions involving the tendency toward fat-dense food and irregular sleep factors resulted in higher WHR and WC measurements. Moreover, 21 significant interactions between the risk allele “C” of the *FTO* rs1421085 and random eating patterns, a tendency toward fat-dense food, irregular sleep, and shift work were observed to increase obesity-related traits such as BMI, WC, HC, WHR, WHtR, and BF%. Regarding the *TMEM18* rs7561317 variant, interactions of the risk allele “G” with random eating patterns, tendency toward fat-dense food, and irregular sleep significantly increased all obesity traits, including BMI, WC, HC, WHR, WHtR, and BF%. Interactions between the *TMEM18* variant and shift work and inadequate sleep were also significantly associated with higher obesity traits in this study. Lastly, researchers examined the *NEGR1* rs2815752 variant and found 24 interactions between the risk allele “G” and random eating patterns, the tendency toward fat-dense food, inadequate and irregular sleep, and shift work, which increased all obesity parameters significantly.^64^

Concerning other lifestyle factors, 1 significant gene–smoking interaction relating to obesity was identified in an investigation of healthy participants from the PROMIS cohort in Pakistan.^63^ This interaction involved the *FLJ33534* rs140133294 variant, which exhibited a positive association with BMI in non-smokers but a negative association in current smokers (Table 3). A separate sample from the same cohort showed a reverse effect of smoking on the gene–BMI association. Several variants (PTBP2 rs11165643, HIP1 rs1167827, GRID1 rs7899106) interacted with smoking to increase BMI; however, these findings were inconclusive.^62^

### Effects of gene–lifestyle interactions on T2D

**Figure 3.**
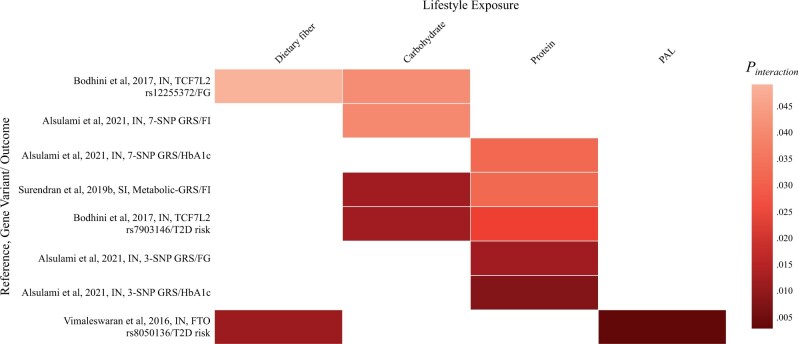
Heat Map Showing the Results of the Significant Interactions Between Gene and Lifestyle Factors in Relation to T2D in Indian and Sinhalese Populations. Alsulami et al (2021)^59^, 7-SNP-GRS=*TCF7L2* (rs12255372, rs7903146); *FTO* (rs8050136, rs918031, rs1588413, rs7193144, rs1076023); Surendran et al (2019b)^68^, metabolic-GRS=*FTO* (rs9939609, rs8050136); *MC4R* (rs17782313, rs2229616); FTO, alpha-ketoglutarate dependent dioxygenase; GRS, genetic risk score; HbA1c, Hemoglobin A1c; SNP, single nucleotide polymorphism; TCF7L2, transcription factor 7-like; *TCF7L2* (rs12255372, rs7903146); *KCNJ11* (rs5219); *CAPN10* (rs3792267, rs2975760, rs5030952); Bodhini et al (2017)^56^; Vimaleswaran et al (2016).^25^ Abbreviations: FG, fasting glucose; FI, fasting insulin; IN, Indian; PAL, physical activity level; SI, Sinhalese; T2D; type 2 diabetes

The current study identified 10 publications that examined the effects of G–L interactions on T2D-related traits such as FG, FI, and HbA1C, in which 12 significant effects of interactions were reported. Findings relating to effects of G–L interactions on T2D, including *P*_interaction_, are shown in Table 5,^25^^,^^56^  Table 6,^59^^,^^68^ and Figure 3.^25^^,^^56^^,^^59^^,^^68^

**Table 5.** Summary Table of Significant Effects of SNP–Lifestyle Interactions on T2D Outcomes

Gene and SNP	Reference	Study design	Population (cohort)/sample size	Lifestyle exposure	T2D outcome	Impact on outcome measures	*P*_interaction_
*FTO* (Alpha-ketoglutarate dependent dioxygenase)							
rs8050136	Vimaleswaran et al (2016)^25^	C-S	Indian (CURES)/1618	PAL (sedentary, moderate, vigorous)	T2D risk	ND	.003
				Dietary fiber intake (g) ([T2D; men: 28.62 ± 0.54, women: 34.91 ± 0.69], [NGT; men: 29.84 ± 0.42, women: 35.73 ± 0.56])	T2D risk	ND	.01
*TCF7L2* (Transcription factor 7-like 2)							
rs7903146	Bodhini et al (2017)^56^	C-S	Indian (CURES)/1682	Carbohydrate (g) (NGT: 419.69 ± 112.33, T2D: 399.96 ± 147.98 [g])	T2D risk	ND	.011
				Protein (g) (NGT: 73.82 ± 20.97, T2D: 69.93 ± 24.56 [g])	T2D risk	ND	.024
rs12255372				Carbohydrate (g) (NGT: 419.69 ± 112.33, T2D: 399.96 ± 147.98 [g])	FG	ND	.041
				Dietary fiber (g) (NGT: 31.41 ± 9.83, T2D: 31.66 ± 12.19 [g])	FG	ND	.049

Outcome measures include diabetes-related traits; ↓ indicates a significant decrease in outcome measure; ↑ indicates a significant increase in outcome measure; ND indicates non-defined impact. Abbreviations: SNP, single nucleotide polymorphism; C-S, cross-sectional; CURES, Chennai Urban Rural Epidemiology Study; FG, fasting glucose; NGT, normal glucose tolerance subjects; PAL, physical activity level (high, medium, low/sedentary); T2D, type 2 diabetes.

**Table 6.** Summary Table of Significant Effects of GRS–Lifestyle Interactions on T2D Outcomes

GRS and SNPs	Reference	Study design	Population (cohort)/sample size	Lifestyle exposure	T2D outcome	Impact on outcome measures	*P*_interaction_
7-SNP GRS: *TCF7L2* rs12255372 and rs7903146; *FTO* rs8050136, rs918031, rs1588413, rs7193144, and rs1076023	Alsulami et al (2021)^59^	C-S	Indian (CURES)/1062	Protein energy % (11 ± 1%)	HbA1c	↑	.032
3-SNP GRS: *TCF7L2* rs12255372 and rs7903146; *FTO* rs8050136				Carbohydrate energy % (65 ± 6%)	FI	↑	.04
				Protein energy % (11 ± 1%)	HbA1c	↑	.007
					FG	↑	.011
Metabolic-GRS: *FTO* rs9939609 and rs8050136; *MC4R* rs17782313 and rs2229616; *TCF7L2* rs12255372 and rs7903146; *KCNJ11* rs5219; *CAPN10* rs3792267, rs2975760, and rs5030952	Surendran, et al (2019b)^68^	C-S	Sinhalese (GOOD)/109	Carbohydrate energy % (69.62 ± 8.80%)	FI	ND	.011
				Protein energy % (11.29 ± 2.31%)	FI	ND	.032

Outcome measures include diabetes-related traits; ↓ indicates a significant decrease in outcome measure; ↑ indicates a significant increase in outcome measure; ND indicates non-defined impact. Abbreviations: GRS, genetic risk score; SNP, single nucleotide polymorphism; HbA1C, Hemoglobin A1c; *CAPN10*, Calpain; C-S, cross-sectional; CURES, Chennai Urban Rural Epidemiology Study; FG, fasting glucose; FI, fasting insulin; *FTO*, alpha-ketoglutarate dependent dioxygenase; GOOD, Genetic of Obesity and Diabetes; *KCNJ11*, Potassium Inwardly Rectifying Channel Subfamily J Member 11; *MC4R*, melanocortin-4-receptor; T2D, type 2 diabetes; *TCF7L2*, transcription factor 7-like 2.

#### Effects of interactions between genes and dietary intake on T2D traits

Effects of gene–diet interactions on T2D were reported in 4 included studies.^25^^,^^56^^,^^59^^,^^68^ In a sample of 1062 Indians recruited from the Chennai Urban Rural Epidemiology Study (CURES) cohort, a significant effect was noted of an interaction between 7-SNP-GRS (*TCF7L2* and *FTO* SNPs) and carbohydrate energy intake (%) (65 ± 6% total energy intake [TEI]) on FI, which can be seen in Table 6.^59^ Carbohydrate intake was also found to significantly interact with the *TCF7L2* rs7903146 variant to modify FG and T2D risk in another sample (*n* = 1682) from the same cohort (CURES).^56^ Additionally, with a GRS approach, cross-sectional data from a sample of 109 healthy Sinhalese adults reported a significant effect of an interaction of a 10-SNP metabolic-GRS (*FTO*, *MC4R*, *TCF7L2*, *KCNJ11*, and *CAPN10* variants) with carbohydrate energy intake (%) (69.62 ± 8.80% TEI) and with protein energy intake (%) (11.29 ± 2.31% TEI) on FI.^68^ Moreover, the influence of protein energy intake on gene–T2D associations was identified in a cross-sectional study that included 1062 adult Indians, where a positive effect of interaction between a 3-SNP GRS (*TCF7L2* and *FTO* SNPs) and total protein intake (%) on FG and HbA1c was reported.^59^ In addition, low plant protein intake (<39 g/day) was associated with increased HbA1c and FG among carriers of >1 risk allele compared with individuals with ≤1 risk allele. Additionally, a higher intake of animal protein (>19 g/day) was associated with greater FG and HbA1c in those with >1 risk allele than in individuals with ≤1 risk allele.^59^ Furthermore, the same study reported a significant effect of an interaction between the 7-SNP GRS and protein intake (%) on HbA1c. Another protein-intake-related interaction was observed with the *TCF7L2* SNP rs7903146 on T2D risk in a cross-sectional study including 861 T2D and 821 NGT Indian subjects.^56^

Two cross-sectional studies from India reported effects of gene–diet interactions on T2D traits involving dietary fiber.^25^^,^^56^ First, an effect of an interaction between the *TCF7L2* SNP rs12255372 and total dietary fiber intake (g) on FG was identified in a sample of 1682 adults recruited from the CURES study; however, this trend was not observed between the SNP rs7903146 and dietary fiber intake on T2D risk.^56^ Another significant effect of an interaction between the *FTO* SNP rs8050136 and dietary fiber intake (g) (32 ± 11 g/day) on T2D was identified in a sample of 566 T2D cases and 496 NGT Indian adults.^25^ However, this interaction was not observed for *FTO* SNP rs11076023. Nevertheless, investigations of fat intake, alcohol consumption, and a vegetarian diet showed no significant effects of interaction with any SNPs or GRSs on T2D-related outcomes in the studies from India.^25^^,^^55^^,^^56^^,^^58^^,^^59^ The effect of the gene–diet interaction on T2D risk has also been explored in a sample of 598 Asian Indian diasporas recruited from the Singapore National Health Survey Cohort; however, researchers did not identify any significant effects of interactions between the *PLIN* SNP rs894160 and dietary exposures on T2D risk in this population.^66^

#### Effects of interactions between genes and physical activity on T2D traits

Across the included articles, 3 cross-sectional studies explored the effects of gene–PA interactions on T2D-related traits in the SA population.^25^^,^^58^^,^^68^ Nevertheless, only 1 reported a significant interaction where PA levels were found to modify the association between the *FTO* SNP rs8050136 and T2D risk in a sample of 734 T2D patients and 884 NGT participants from India; however, this effect was not shown for the *FTO* SNP rs11076023.^25^ The remaining studies did not identify significant effects of interactions between PA and GRSs on any T2D-related outcomes in the Indian or Sinhalese population.^58^^,^^68^

## DISCUSSION

This is the first systematic review summarizing all of the evidence of G–L interactions and their impacts on metabolic diseases, including obesity and T2D, in the SA population. Seven of the 15 identified publications reported statistically significant effects of G–L interactions on obesity or T2D traits^25^^,^^56^^,^^59^^,^^61^^,^^63^^,^^64^^,^^68^ (Figures 2 and 3). Several studies suggested that the *FTO* and *TCF7L2* variants were the most robust genetic predictors for obesity and T2D in SA, respectively^26^^,^^69–71^ which is similar to the findings in European research.^72^^,^^73^ Therefore, variants of the *FTO* and *TCF7L2* genes were the most frequently examined variants (alone or aggregated) across studies in the current review (in 11 and 6 studies, respectively). Most studies found that the significant effects of interactions were on obesity-related parameters, including BMI, WC, and WHR (Tables 3 and 4), while a limited number of reported interactions were related to T2D outcomes (Tables 5 and 6).

The current findings from the SA-origin population have demonstrated that multifactorial metabolic diseases, including obesity and diabetes, are affected by the interaction between genetic and lifestyle factors. In particular, the variants of *FTO* and the *TCF7L2* have been found to have significant interactions with dietary intake, including carbohydrates, protein, and dietary fiber, in terms of T2D risk in the SA population.^25^^,^^56^^,^^59^^,^^68^ This is corroborated by data from the Nurses’ Health Study (NHS), which indicated that the risk of T2D associated with *TCF7L2* was increased by the quality and quantity of carbohydrates in the diet of diabetic American women.^74^ Furthermore, interactions involving high carbohydrate intake were observed to enhance obesity traits, resulting in an increased risk of obesity. In contrast, the current findings indicated that a higher dietary fiber intake may have an attenuating influence on metabolic disease risk as determined by genetic factors.^25^^,^^68^ A similar moderating effect of fiber-gene interaction on T2D was also observed across various populations.^41^ Additionally, the current data show that protein and carbohydrate intakes interact with B12-GRS, having a significant effect on metabolic-related traits in Sinhalese populations.^68^ Although there is limited evidence concerning the effect of interaction of fat intake with genes on metabolic disease risk in the SA population, interestingly, high intake of SFA (>23 g/day) was reported to interact with 3SNP-GRS (*CETP rs4783961* and *LPL* rs327, rs3200218), increasing obesity risk in Indians.^61^

In addition to dietary factors, the current findings validate the modifying impact of PA on the association between genes and metabolic traits among SA individuals. Exposure to PA was intensively investigated in the included studies. Low PA levels were shown to interact with several gene variants to significantly increase obesity risk, particularly in the Pakistani population.^62^^,^^64^ Similarly, physical inactivity was found to alter the association of the *FTO* gene variant with T2D in Asian Indians, increasing the risk of the disease.^25^. Consistent findings were shown for the role of PA in attenuating the susceptibility to metabolic traits induced by *FTO* variants in various populations.^26^^,^^29^^,^^75–77^ This effect of PA on the *FTO*–obesity association was also reported by Roden et al^78^ in a multi-ethnic sample of 17 423 participants, of whom 15.8% were SA individuals.

Inadequate sleep and increased tobacco use are further results of the dramatic lifestyle changes that have recently occurred in the South Asian population.^18^ Sleep deprivation and disturbance are proven to be associated with diabetes risk.^18^^,^^79^ For instance, sleep restricted to 4 hours per night has been shown to cause an imbalance in the appetite-regulating hormones leptin and ghrelin.^80^ Short sleep (<7 hours/night) was also identified as an independent risk factor for T2D in Hispanic and Caucasian populations.^81^ Particularly among Asian Indians, studies have shown that snoring, daytime sleepiness, and sleep apnoea are positively and independently associated with glucose intolerance, metabolic syndrome, and FI levels.^82^^,^^83^ Although some studies suggest that the interaction of sleep duration with metabolic-associated variants does not influence diabetes risk,^84^ findings from the included studies indicate that poor sleeping patterns, characterized by irregular sleeping and wake-up timing, inadequate sleep, and shift work, significantly increased obesity risk by interacting with several genetic variants among the Pakistani population.^64^ Although studies have found convincing evidence of the effect of smoking on genetic predisposition to obesity, the direction of the effect on obesity-related traits has been inconsistent.^85^ According to the findings, smoking mitigates the risk of obesity in Pakistanis by interacting significantly with the *FLJ33534* risk allele.^63^

Most of the metabolic disease data were obtained in India, the SA country with the largest diabetes burden^18^^,^^86^; therefore, most identified publications in this study were in the Indian population (67%),^25^^,^^55–61^^,^^66^^,^^67^ particularly from the CURES cohort.^25^^,^^56–61^ The current systematic search did not identify any interaction studies from Afghanistan, Bangladesh, Bhutan, Maldives, or Nepal, only studies from India, Pakistan, and Sri Lanka. Consequently, this may have led to an under-representation of other countries in the SA subregion. Furthermore, the high heterogeneity of the studies caused by the wide variation in the investigated lifestyle factors, genetic variants, and outcomes limited the conduction of a meta-analysis.

One challenging task for nutrigenetic studies is replicating the G–L interaction effects^63^^,^^87^; this review indicates that most G–L interaction data in the SA population have yet to be replicated. Another challenge in this study is the increased likelihood of false-positive findings arising from testing for interactions across multiple genetic variants with lifestyle variables, generating multiple comparisons.^88^ However, this can be controlled by including correction for multiple testing approaches, such as the Bonferroni correction.^40^^,^^89^ In this study, fewer than half of the included studies confirmed a correction for multiple comparisons in their methodology (Table S4). Another methodological concern of nutrigenetic studies is low statistical power, which may limit the chance of detecting an actual interaction.^41^ Hence, a large sample size of thousands of participants is needed to identify significant effects of interactions and diminish the probability of underpowered analysis in such studies,^34^^,^^89^ especially for investigating multifactorial diseases with a slight genetic main effect.^90^ It has been estimated that a minimum sample size of around 6500 participants is required to reach 80% power to detect the G–L interaction effects in a case–control study design.^91^ In this review, approximately 70% of the studies had a sample size of <2000 participants, and only 2 studies exceeded 10 000 participants.^62^^,^^63^ Moreover, most studies (47%) did not provide any information regarding power and sample size calculations for the interaction analysis,^25^^,^^55^^,^^58^^,^^59^^,^^61^^,^^64^^,^^65^ while 20% stated incapability to perform power calculations.^56^^,^^57^^,^^68^

Notably, FFQs and self-reporting questionnaires were the only tools used for lifestyle exposure assessment in the identified studies, which could be justified by their low cost, feasibility, and suitability for prospective and retrospective study designs.^92^^,^^93^ However, self-reported assessment instruments are subject to substantial error and misreporting, which generates bias, decreases measurement accuracy, and consequently affects the findings.^39^^,^^92–96^ This is considered a crucial concern in G–L studies.^41^ Two studies included in this review did not state whether the questionnaire was validated.^55^^,^^64^ Additionally, it is important to consider the utilization of population-specific dietary scores, such as the Indian Diet Quality Score (IDQS), to accurately evaluate the risk of metabolic disease in this population.^97^ Furthermore, 80% of the studies included in this review used a cross-sectional design; hence, recall bias could be a potential problem, especially for studies exploring lifestyle influences on conditions such as obesity and T2D, since lifestyle exposure and outcome were investigated concurrently.^36^^,^^98^

The lack of replication and inconsistency in the findings across the studies could be due to the small sample size, imprecise measurements of dietary and lifestyle exposures, and low statistical power of the current studies. Although large population-based prospective cohorts such as the UK Biobank have provided robust lifestyle measurements,^99–102^ this is still challenging for the SA population due to the limited number of studies with large sample sizes.^34^ However, some studies suggested that sufficient statistical power could be achieved by investing in repeated, more precise exposure measurements, which is considered more appropriate and cost-effective than increasing the sample size.^103^^,^^104^ Moreover, combining the effect of multiple SNPs using a GRS approach is recommended to avoid loss of power due to multiple testing, allowing for G–L interactions to be detected.^105^^,^^106^

In this context, it is also consequential to consider the application of artificial intelligence and machine-learning approaches when planning for G–L interaction studies, as the evolution of these approaches has gained wider scientific attention. Machine learning and mathematical modelling techniques have been applied in nutritional epidemiological studies for evaluating and predicting disease risks such as obesity,^107–110^ identifying genetic variants and dietary and G–L factors that can be significant indicators of disease,^108^ objectively collecting dietary and PA data,^111–114^ and driving dietary recommendations.^115^ This is achieved by integrating plasma, microbiome, anthropometric, dietary, and lifestyle data into a system that learns patterns within the dataset and applies these patterns to predicting the metabolic outcome.^38^^,^^42^^,^^113^ Therefore, employing advanced machine-learning approaches and analytical methods is a substantial step forward for nutrigenetic studies.

This is the first study to systematically review and analyze the effects of G–L interaction on metabolic-disease-related outcomes in the SA population. Moreover, in addition to the standardized ROBINS-I and AXIS tools, a quality score designed explicitly for G–L studies was applied to evaluate the methodological quality of the included studies.^41^^,^^54^ However, some limitations need to be addressed. Given the small number of studies and the variation in the investigated lifestyle factors, genetic variants, and outcomes, conducting a meta-analysis was infeasible. Another important consideration was that most included studies originated from India, which may limit the generalizability of the evidence to the broader SA population. However, most identified interactions were derived from small-scale studies, without correction for multiple testing, focusing mainly on Indian, Pakistani, and Sinhalese people. Furthermore, most findings are yet to be replicated in more extensive trials.

## CONCLUSION

In conclusion, this systematic review revealed various G–L interactions that modify metabolic disease risk in the SA population. The findings of this study indicated that exposure to unhealthy lifestyle factors such as low PA, inadequate sleep, and irregular sleeping patterns prompt susceptibility to obesity and T2D risk determined by genetic factors. Additionally, higher dietary consumption of carbohydrates, protein, and fat has been shown to influence metabolic risk in this population through interactions with genetic variations. Consequently, adopting healthy dietary patterns, which include higher dietary fiber intake and increased PA, may offer advantages to individuals with an increased risk of metabolic diseases due to genetic predisposition. It has been suggested that lifestyle interventions might yield particularly good results among individuals at high risk for T2D. While the evidence is of low certainty, it suggests inter-individual variability in how people respond to these interventions.^116^ Hence, establishing large longitudinal quantitative trait studies with precise lifestyle exposure measurements focusing on macro- and micronutrients is pivotal to understanding the effects of the interplay between genetic and lifestyle factors on metabolic diseases in such high-risk populations. Future G–L interaction research should shed light on other parts of the southern subregion of Asia, as current evidence is mainly from India and Pakistan, allowing the future development of personalized prognostic and prevention strategies for obesity and T2D in SA, especially in the face of the current metabolic disease epidemic.

## Supplementary Material

nuae115_Supplementary_Data

## Notes

This article is part of a Special Collection on Precision Nutrition.
