# Supplementary material for: A Systematic Review of the Effect of Gene–Lifestyle Interactions on Metabolic-Disease-Related Traits in South Asian Populations
Source: Nutr Rev. 2024 Sep 16;83(6):1061–82. doi: 10.1093/nutrit/nuae115 (PMC12066952; doi:10.1093/nutrit/nuae115)
Supplement: nuae115_Supplementary_Data [file nuae115_supplementary_data.zip › nuae115_Supplementary_Data/Bineid et al_Supplementary file 2.docx]

**Supplementary Materials**

**Table S 1:** Search Strings in Different Databases

| **MEDLINE (PubMed)** |
| --- |
| ((“South Asia*” [tiab] OR Bangladesh* [tiab] OR Pakistan* [tiab] OR Sri Lanka [tiab] OR Sinhalese [tiab] OR Bhutan* [tiab] OR Maldiv* [tiab] OR India* [tiab] OR Nepal* [tiab] OR and Afghan* [tiab]) AND (diet OR “dietary pattern*” OR nutrition OR carb* OR fat* OR lipid* OR fatty acid* OR glucose OR fibre OR fiber OR sugar* OR “total energy intake” OR “energy intake” TEI OR SFA* OR “saturated fatty acid*” OR PUFA* OR “polyunsaturated fatty acid*” OR MUFA* OR “monounsaturated fatty acid*” OR “trans fat*” OR protein* OR “amino acid*” OR “dietary fibre” OR micronutrient* OR macronutrient* OR “soluble fibre” OR “soluble fiber” OR “insoluble fibre” OR “insoluble fiber” OR caffeine OR coffee OR alcohol OR “Physical activity” OR lifestyle OR “life style” OR exercise OR sport* OR “physical inactivity” OR “physically inactive” OR sedentary) AND (SNP* OR “single nucleotide polymorphism” OR “genetic variation” OR allele OR “genetic risk score” OR GRS OR “polygenic risk score” OR PRS OR chromosome OR exon OR homozygote OR heterozygote OR “risk allele” OR “gene-diet interaction” OR “gene-lifestyle interaction” OR “gene-physical activity interaction” OR “gene-nutrient interaction” OR "gene nutrient" [tiab:~5] OR "gene diet" [tiab:~5] OR "gene lifestyle" [tiab:~5] OR "gene physical" [tiab:~5] OR nutrigenetic*) AND (diabetes OR T2D OR “type 2 diabetes” OR HbA1c OR “fasting glucose” OR “fasting insulin” OR glycated OR H?emoglobin OR haemoglobin OR “insulin resistance” OR “gestational diabetes” OR MODY OR “Maturity onset diabetes of the Young” OR “glucose tolerance” OR Obesity OR WHR OR “waist hip ratio” OR WC OR “waist circumference” OR HC OR “hip circumference” OR BMI OR “body mass index” OR adiposity OR “body fat” OR “body composition” OR “Metabolic disease” OR CVD OR cardiovascular OR LDL OR HDL OR VLDL OR LDL-c OR HDL-c OR VDL-c OR “low density lipoprotein” OR “high density lipoprotein” OR “very low-density lipoprotein” OR “blood lipid profile” OR “plasma lipid profile” OR “myocardial infarction” OR homocysteine OR HCY OR chylomicron*)) |
| ((diet* OR nutrient* OR nutrition OR macronutrient* OR "fat intake" OR "fatty acid intake" OR "fat quality" OR "saturated fat*" OR SFA OR fat* OR "monounsaturated fat*" OR MUFA OR "polyunsaturated fat" OR PUFA OR "unsaturated fat" OR animal fat OR oil* OR carbohydrate* OR fibre OR fiber OR grain* OR "glycaemic index" OR "glycaemic load" OR "glycemic index" OR "glycemic load" OR sugar OR protein* OR "dietary protein" OR "plant-based protein" OR "animal protein" OR micronutrient* OR mineral* OR magnesium OR zinc OR iron OR vitamin* OR vitamin D OR meal* OR meat OR nuts OR fruit* OR vegetable* OR alcohol OR coffee OR "sugar-sweetened beverages" OR SSB OR "dietary pattern*" OR "dietary habit*" OR "dietary change*" OR "traditional pattern" OR "western diet" OR "westernized diet" OR "western pattern" OR diet[MeSH Terms] OR meals[MeSH Terms] OR cooking[MeSH Terms] OR nutritive value[MeSH Terms] OR dietary carbohydrate[MeSH Terms] OR meat[MeSH Terms] OR dietary fiber[MeSH Terms] OR nuts[MeSH Terms] OR fatty acids[MeSH Terms] OR dietary fats[MeSH Terms] OR acids, trans fatty[MeSH Terms] OR dietary fats, unsaturated[MeSH Terms] OR fatty acids, omega 3[MeSH Terms] OR fatty acids, omega 6[MeSH Terms] OR alcohol[MeSH Terms] OR coffee[MeSH Terms] OR magnesium[MeSH Terms] OR zinc[MeSH Terms] OR vitamin d[MeSH Terms] OR 1,25 dihydroxy 20 epi vitamin d3[MeSH Terms] OR diet, high fat[MeSH Terms] OR micronutrient[MeSH Terms] AND (interact* OR modif* OR modulat*) AND (South Asia OR Pakistan* OR Sri Lanka OR Sinhalese* OR Singhalese OR Bangladesh) AND (gene-nutrient interaction* OR nutrigenetics OR gene OR genotype OR genetic* OR SNP OR allele* OR polymorphism OR genetic risk score OR GRS OR genes[MeSH Terms] OR genotype[MeSH Terms] OR genetic variation[MeSH Terms] OR copy number polymorphism[MeSH Terms] OR nucleotide polymorphism, single[MeSH Terms] OR alleles[MeSH Terms] OR nutrigenetics[MeSH Terms]))) AND (diabetes) |
| ((diet* OR nutrient* OR nutrition OR macronutrient* OR "fat intake" OR "fatty acid intake" OR "fat quality" OR "saturated fat*" OR SFA OR fat* OR "monounsaturated fat*" OR MUFA OR "polyunsaturated fat" OR PUFA OR "unsaturated fat" OR animal fat OR oil* OR carbohydrate* OR fibre OR fiber OR grain* OR "glycaemic index" OR "glycaemic load" OR "glycemic index" OR "glycemic load" OR sugar OR protein* OR "dietary protein" OR "plant-based protein" OR "animal protein" OR micronutrient* OR mineral* OR magnesium OR zinc OR iron OR vitamin* OR vitamin D OR meal* OR meat OR nuts OR fruit* OR vegetable* OR alcohol OR coffee OR "sugar-sweetened beverages" OR SSB OR "dietary pattern*" OR "dietary habit*" OR "dietary change*" OR "traditional pattern" OR "western diet" OR "westernized diet" OR "western pattern" OR diet[MeSH Terms] OR meals[MeSH Terms] OR cooking[MeSH Terms] OR nutritive value[MeSH Terms] OR dietary carbohydrate[MeSH Terms] OR meat[MeSH Terms] OR dietary fiber[MeSH Terms] OR nuts[MeSH Terms] OR fatty acids[MeSH Terms] OR dietary fats[MeSH Terms] OR acids, trans fatty[MeSH Terms] OR dietary fats, unsaturated[MeSH Terms] OR fatty acids, omega 3[MeSH Terms] OR fatty acids, omega 6[MeSH Terms] OR alcohol[MeSH Terms] OR coffee[MeSH Terms] OR magnesium[MeSH Terms] OR zinc[MeSH Terms] OR vitamin d[MeSH Terms] OR 1,25 dihydroxy 20 epi vitamin d3[MeSH Terms] OR diet, high fat[MeSH Terms] OR micronutrient[MeSH Terms] AND (interact* OR modif* OR modulat*) AND (South Asia OR Pakistan* OR Sri Lanka OR Sinhalese* OR Singhalese OR Bangladesh) AND (gene-nutrient interaction* OR nutrigenetics OR gene OR genotype OR genetic* OR SNP OR allele* OR polymorphism OR genetic risk score OR GRS OR genes[MeSH Terms] OR genotype[MeSH Terms] OR genetic variation[MeSH Terms] OR copy number polymorphism[MeSH Terms] OR nucleotide polymorphism, single[MeSH Terms] OR alleles[MeSH Terms] OR nutrigenetics[MeSH Terms]))) AND (obes* OR BMI OR Adipos*) |
| ((Behavior, Sedentary[MeSH Terms] OR Sedentary Behaviors[MeSH Terms] OR Sedentary Lifestyle[MeSH Terms] OR Lifestyle, Sedentary[MeSH Terms] OR Physical Inactivity[MeSH Terms] OR Inactivity, Physical[MeSH Terms] OR Lack of Physical Activity[MeSH Terms] OR Sedentary Time[MeSH Terms] OR Sedentary Times[MeSH Terms] OR Time, Sedentary[MeSH Terms] Or Exercises Physical Activity OR Activities, Physical OR Activity, Physical OR Physical Activities OR Exercise, Physical OR Exercises, Physical OR Physical Exercise OR Physical Exercises OR Acute Exercise OR Acute Exercises OR Exercise, Acute OR Exercises, Acute OR Exercise, Isometric OR Exercises, Isometric OR Isometric Exercises OR Isometric Exercise OR Exercise, Aerobic OR Aerobic Exercise OR Aerobic Exercises OR Exercises, Aerobic OR Exercise Training OR Exercise Trainings OR Training, Exercise OR Trainings, Exercise) AND (South Asia* OR Pakistan* OR Sri Lanka OR Sinhalese* OR Singhalese OR Bangladesh* OR Bhutan OR afghanistan OR nepal* OR maldives) AND (gene-nutrient interaction* OR nutrigenetics OR gene OR genotype OR genetic* OR SNP OR allele* OR polymorphism OR genetic risk score OR GRS OR genes[MeSH Terms] OR genotype[MeSH Terms] OR genetic variation[MeSH Terms] OR copy number polymorphism[MeSH Terms] OR nucleotide polymorphism, single[MeSH Terms] OR alleles[MeSH Terms] OR nutrigenetics[MeSH Terms])) AND (obes* OR BMI OR Adipos*) |
| ((Behavior, Sedentary[MeSH Terms] OR Sedentary Behaviors[MeSH Terms] OR Sedentary Lifestyle[MeSH Terms] OR Lifestyle, Sedentary[MeSH Terms] OR Physical Inactivity[MeSH Terms] OR Inactivity, Physical[MeSH Terms] OR Lack of Physical Activity[MeSH Terms] OR Sedentary Time[MeSH Terms] OR Sedentary Times[MeSH Terms] OR Time, Sedentary[MeSH Terms] Or Exercises Physical Activity OR Activities, Physical OR Activity, Physical OR Physical Activities OR Exercise, Physical OR Exercises, Physical OR Physical Exercise OR Physical Exercises OR Acute Exercise OR Acute Exercises OR Exercise, Acute OR Exercises, Acute OR Exercise, Isometric OR Exercises, Isometric OR Isometric Exercises OR Isometric Exercise OR Exercise, Aerobic OR Aerobic Exercise OR Aerobic Exercises OR Exercises, Aerobic OR Exercise Training OR Exercise Trainings OR Training, Exercise OR Trainings, Exercise) AND (South Asia* OR Pakistan* OR Sri Lanka OR Sinhalese* OR Singhalese OR Bangladesh* OR Bhutan OR afghanistan OR nepal* OR maldives) AND (gene-nutrient interaction* OR nutrigenetics OR gene OR genotype OR genetic* OR SNP OR allele* OR polymorphism OR genetic risk score OR GRS OR genes[MeSH Terms] OR genotype[MeSH Terms] OR genetic variation[MeSH Terms] OR copy number polymorphism[MeSH Terms] OR nucleotide polymorphism, single[MeSH Terms] OR alleles[MeSH Terms] OR nutrigenetics[MeSH Terms])) AND (diabet* OR T2D OR type 2 diabetes OR HbA1c OR insulin*) |

| **SCOPUS** |
| --- |
| (TITLE-ABS-KEY(South Asia) AND ALL(" gene diet interaction" OR "gene lifestyle interaction" OR SNP* OR single nucleotide polymorphism) AND ALL(diet OR carb OR fat OR lipid OR "fatty acid" OR fibre OR fiber OR "total energy intake" OR protein OR micronutrient OR macronutrient OR "Physical activity" OR lifestyle OR exercise) AND ALL(diabetes OR T2D OR "type 2 diabetes" OR HbA1c OR "fasting glucose" OR "fasting insulin" OR "insulin resistance" OR Obesity OR "waist hip ratio" OR "waist circumference" OR BMI OR "body mass index" OR "Metabolic disease" OR CVD OR cardiovascular OR "lipid profile")) |
| ( TITLE-ABS-KEY ( pakistan  OR  india  OR  "Sri Lanka"  OR  bangladesh  OR  nepal  OR  afghanistan  OR  bhutan  OR  maldives )  AND  ALL ( " gene diet interaction"  OR  "gene lifestyle interaction"  OR  snp*  OR  single  AND nucleotide  AND polymorphism )  AND  ALL ( diet  OR  carb  OR  fat  OR  lipid  OR  "fatty acid"  OR  fibre  OR  fiber  OR  "total energy intake"  OR  protein  OR  micronutrient  OR  macronutrient  OR  "Physical activity"  OR  lifestyle  OR  exercise )  AND  ALL ( diabetes  OR  t2d  OR  "type 2 diabetes"  OR  hba1c  OR  "fasting glucose"  OR  "fasting insulin"  OR  "insulin resistance"  OR  obesity  OR  "waist hip ratio"  OR  "waist circumference"  OR  bmi  OR  "body mass index"  OR  "Metabolic disease"  OR  cvd  OR  cardiovascular  OR  "lipid profile" ) ) |
| **Web of Science** |
| South Asia OR Bangladesh* OR Pakistan* OR Sri Lanka OR Bhutan* OR Maldiv* OR India* OR Nepal* OR Afghan* (Topic) and diet OR carb* OR fat* OR lipid* OR fatty acid* OR glucose OR fiber OR sugar* OR total energy intake OR TEI OR protein* OR amino acid* OR micronutrient* OR macronutrient* OR caffeine OR coffee OR alcohol OR Physical activity OR lifestyle OR exercise (All Fields) and SNP* OR single nucleotide polymorphism OR genetic variation OR genetic risk score OR GRS OR chromosome OR homozygote OR heterozygote OR nutrigenetic* OR gene-diet interaction OR gene-lifestyle interaction OR gene-physical activity interaction OR gene-nutrient interaction (All Fields) and diabetes OR T2D OR type 2 diabetes OR HbA1c OR fasting glucose OR fasting insulin OR insulin resistance OR gestational diabetes OR glucose tolerance OR Obesity OR WHR OR waist hip ratio OR WC OR waist circumference OR BMI OR body mass index OR adiposity OR body fat OR Metabolic disease OR blood lipid profile OR plasma lipid profile (All Fields) |
| **Search strings to be used in PubMed, Web of Science and Scopus** |
| (((nutrigenetic*) AND (interact*)) AND (india*)) AND (diabetes) |
| (((nutrigenetic*) AND (interact*)) AND (pakistan*)) AND (diabetes) |
| (((nutrigenetic*) AND (interact*)) AND (sri lanka*)) AND (diabetes) |
| (((nutrigenetic*) AND (interact*)) AND (sinhalese*)) AND (diabetes) |
| (((nutrigenetic*) AND (interact*)) AND (bangladesh*)) AND (diabetes) |
| ((((gene*) AND (diet*)) AND (interact*)) AND (diabetes)) AND (india*) |
| Gene* AND diet* AND interact* AND diabetes AND pakistan* |
| Gene* AND diet* AND interact* AND diabetes AND lanka* |
| Gene* AND diet* AND interact* AND diabetes AND bangladesh* |
| (ALL=(nutrigenetic*)) AND ALL=(india*) |
| ((ALL=(gene-diet)) AND ALL=(interaction)) AND ALL=(india*) |
| ((ALL=(gene-nutrient)) AND ALL=(interaction)) AND ALL=(india*) |
| (ALL=(nutrigenetic*)) AND ALL=(pakistan*) |
| ((ALL=(gene-nutrient)) AND ALL=(interaction)) AND ALL=(pakistan*) |
| ((ALL=(gene-diet)) AND ALL=(interaction)) AND ALL=(pakistan*) |
| (ALL=(nutrigenetic*)) AND ALL=(sri lanka*) |
| ((ALL=(gene-diet)) AND ALL=(interaction)) AND ALL=(sri lanka*) |
| ((ALL=(gene-nutrient)) AND ALL=(interaction)) AND ALL=(sri lanka*) |
| (ALL=(nutrigenetic*)) AND ALL=(Sinhalese*) |
| (ALL=(nutrigenetic*)) AND ALL=(Bangladesh*) |
| (((nutrigenetic*) AND (interact*)) AND (india*)) AND (obes* OR BMI OR adiposity) |
| (((nutrigenetic*) AND (interact*)) AND (india*)) AND (obes* and BMI) |
| (((nutrigenetic*) AND (interact*)) AND (pakistan*)) AND (obes* OR BMI OR adiposity) |
| (((nutrigenetic*) AND (interact*)) AND (Lanka*)) AND (obes* OR BMI OR adiposity) |
| (((nutrigenetic*) AND (interact*)) AND (sinhalese*)) AND (obes* OR BMI OR adiposity) |
| (((nutrigenetic*) AND (interact*)) AND (bangladesh*)) |
| (((nutrigenetic*) AND (interact*)) AND (bhutan*)) |
| (((nutrigenetic*) AND (interact*)) AND (maldives*)) |
| (((nutrigenetic*) AND (interact*)) AND (nepal*)) |
| (((nutrigenetic*) AND (interact*)) AND (afghanistan*)) |
| (((gene-diet*) AND (india*))) |
| (((gene-nutrient*) AND (india*))) |
| (((gene-diet*) AND (pakistan*))) |
| (((gene-nutrient*) AND (pakistan*))) |
| (((gene-diet*) AND (lanka*))) |
| (((gene-nutrient*) AND (lanka*))) |
| (((gene-diet*) AND (bangladesh*))) |
| (((gene-nutrient*) AND (bangladesh*))) |
| (((gene-diet*) AND (bhutan*))) |
| (((gene-nutrient*) AND (bhutan))) |
| (((gene-diet*) AND (maldives*))) |
| (((gene-nutrient*) AND (maldives))) |
| (((gene-diet*) AND (afghanistan*))) |
| (((gene-nutrient*) AND (afghanistan))) |
| Gene* AND physical activity AND India* |
| ((gene*[Title/Abstract]) AND (physical activity[Title/Abstract]) AND (pakistan*[Title/Abstract]) AND (interact*)) |
| ((gene*[Title/Abstract]) AND (physical activity[Title/Abstract]) AND (lanka*[Title/Abstract]) AND (interact*)) |
| ((gene*[Title/Abstract]) AND (physical activity[Title/Abstract]) AND (bangladesh*[Title/Abstract]) AND (interact*)) |

**Table S 2:** Assessment using the Risk of Bias in Non-Randomized Studies – of Interventions (ROBINS-I)

|  | **Answer** | **[Description]** |  | **[Description]** |  | **Description** |
| --- | --- | --- | --- | --- | --- | --- |
| **Bias due to confounding** | **Ahmed et al., 2015** |  | **Jyothi & Reddy., 2015** |  | **Rana et al., 2021** |  |
| 1.1 Is there potential for confounding of the effect of exposure in this study? If N or PN to 1.1: the study can be considered to be at low risk of bias due to confounding and no further signalling questions need be considered Y / PY / PN / N | PN | The researchers used validates tools by trained officers; applied correction for multiple testing and all models had been adjusted for potential confounders. | PN | Representative and homogeneous sample from Hyderabad India; adjusted for covariates; applied Bonferroni correction for multiple testing. | PN | Validated measurement tools was used, correction for multiple comparison was performed, and potential confounders were adjuster for. |
| If Y/PY to 1.1, answer 2.1 and 1.3 to determine whether there is a need to assess time-varying confounding: |  |  |  |  |  |  |
| 1.2. If Y or PY to 1.1: Was the analysis based on splitting, follow up time according to exposure received? |  |  |  |  |  |  |
| If N or PN to 1.2, answer questions 1.4 to 1.6, which relate to baseline confounding |  |  |  |  |  |  |
| 1.3. If Y or PY to 1.2: Were exposure discontinuations or switches likely to be related to factors that are prognostic for the outcome? |  |  |  |  |  |  |
| 1.4. Did the authors use an appropriate analysis method that adjusted for all the critically important confounding areas? |  |  |  |  |  |  |
| 1.6. Did the authors avoid adjusting for post-exposure variables? |  |  |  |  |  |  |
| If Y or PY to 1.3, answer questions 1.7 and 1.8, which relate to time-varying confounding |  |  |  |  |  |  |
| 1.7. Did the authors use an appropriate analysis method that adjusted for all the critically important confounding areas and for time-varying confounding? |  |  |  |  |  |  |
| 1.8. If Y or PY to 1.7: Were confounding areas that were adjusted for measured validly and reliably by the variables available in this study? |  |  |  |  |  |  |
| J**udgment** | **Low** |  | **Low** |  | **Low** |  |
| **Bias in selection of participants into the study** |  |  |  |  |  |  |
| 2.1. Was selection of participants into the study (or into the analysis) based on variables measured after the start of the exposure? | N | Based on previous myocardial infarction | N | Based on T2D diagnosis | N | Based on BMI measurment |
| If N or PN to 2.1 go to 2.4 |  |  |  |  |  |  |
| 2.4 Do start of follow-up and start of exposure coincide for most participants? | N | Participants were already exposed, although the selection criteria were not based on this exposure. | N | Participants were already exposed, although the selection criteria was not based on this exposure. | N | Participants were already exposed, although the selection criteria were not based on this exposure. |
| J**udgment** | **Moderate** |  | **Moderate** |  | **Moderate** |  |
| **Bias in classification of exposures** |  |  |  |  |  |  |
| 3.1 Is exposure status well defined? | N | Physical activity and smokers as 3 categories. | Y |  | Y | All exposures are well-defined |
| 3.2 Did entry into the study begin with start of the exposure? | N | Participants were already exposed, although the selection criteria were not based on this exposure. | N |  | N | Participants were already exposed |
| 3.3 Was information used to define exposure status recorded prior to outcome assessment? | Y |  | Y |  | Y |  |
| 3.4 Could classification of exposure status have been affected by knowledge of the outcome or risk of the outcome? | N |  | N |  | N |  |
| 3.5 Were exposure assessment methods robust (including methods used to input data)? | N | Analysis conducted using categorical variables missing detail | N |  | Y |  |
| J**udgment** | **High** |  | **Moderate** |  | **Moderate** |  |
| **Bias due to departures from intended exposures** |  |  |  |  |  |  |
| 4.1. Is there concern that changes in exposure status occurred among participants? | N |  | N |  | N |  |
| J**udgment** | **Low** |  | **Low** |  | **Low** |  |
| **Bias due missing data** |  |  |  |  |  |  |
| 5.1 Were there missing outcome data? | N |  | N |  | N |  |
| 5.2 Were participants excluded due to missing data on exposure status? | N |  | N |  | N |  |
| 5.3 Were participants excluded due to missing data on other variables needed for the analysis? | Y | Missing rate for genetic data >5 % they were also dropped from the analyses. | N |  | N |  |
| J**udgment** | **Moderate** |  | **Low** |  | **Low** |  |
| **Bias in measurement of outcomes** |  |  |  |  |  |  |
| 6.1 Could the outcome measure have been influenced by knowledge of the exposure received? | N |  | N |  | N |  |
| 6.2 Was the outcome measure sensitive? | Y |  | Y |  | Y |  |
| 6.3 Were outcome assessors unaware of the exposure received by study participants? | N |  | N |  | N |  |
| 6.4 Were the methods of outcome assessment comparable across exposure groups? | Y |  | Y |  | Y |  |
| 6.5 Were any systematic errors in measurement of the outcome unrelated to exposure received? | N |  | N |  | N |  |
| J**udgment** | **Moderate** |  | **Moderate** |  | **Moderate** |  |
| **bias in selection of the reported result** |  |  |  |  |  |  |
| Is the reported effect estimate likely to be selected, on the basis of the results, from... |  |  |  |  |  |  |
| 7.1. …multiple outcome measurements within the outcome domain? | N |  | N |  | N |  |
| 7.2 …multiple analyses of the intervention- outcome relationship? | N |  | N |  | N |  |
| 7.3 ... different subgroups? | N |  | N |  | N |  |
| **Judgment** | **Low** |  | **Low** |  | **Low** |  |

**Table S 3:** Explanation Judgment score for methodical quality of studies.

|  | Low quality | Intermediate quality | High quality |
| --- | --- | --- | --- |
| Interaction as primary study goal | No = -1 | Not known = 0 | Yes = 1 |
| Formal test for interaction | No = -1 | Not known or stratified analysis= 0 | Yes = 1 |
| Correction for multiple testing | No = -1 | Not known = 0 | Yes or not necessary = 1 |
| Correction for ethnicity | No = -1 | Not known = 0 | Yes or not applicable=1 |
| Hardy-Weinberg equilibrium | No or not stated = -1 | - | Yes = 1 |
| Group similarity at baseline tested | No = -1 | Not known = 0 | Yes = 1 |
| Sample size | <1000 = -1 | 1000-5000 = 0 | >5000 = 1 |
| Sufficient details of study procedure stated | No = -1 | - | Yes = 1 |

***Table S 4:*** Judgment score for methodological quality assessments of included studies.

| Study | Interaction as primary study goal | Statistical test for interaction | Correction for multiple testing | Correction for ethnicity | Hardy-Weinberg Equilibrium | Test of group similarity at baseline | Sample size | Sufficient study details | Score |
| --- | --- | --- | --- | --- | --- | --- | --- | --- | --- |
| Corella et al., 2006 | 1 | 1 | -1 | 1 | 1 | 1 | -1 | 1 | 4 |
| Corella et al., 2011 | 1 | 1 | -1 | 1 | 1 | 1 | -1 | 1 | 4 |
| Ahmad et al., 2015 | 1 | 1 | 1 | -1 | 1 | -1 | 1 | 1 | 4 |
| Uma Jyothi & Reddy, 2015 | 1 | 1 | 1 | 1 | -1 | 1 | 0 | 1 | 5 |
| Ahmad et al., 2016 | 1 | 1 | -1 | -1 | 1 | 1 | 1 | 1 | 4 |
| Vimaleswaran et al., 2016 | 1 | 1 | 0 | 0 | 1 | 1 | 0 | 1 | 5 |
| Bodhini et al., 2017 | 1 | 1 | 1 | -1 | -1 | 1 | -1 | 1 | 2 |
| Merritt, Jamnik, & El-Sohemy, 2018 | 1 | 1 | -1 | 1 | -1 | 1 | 0 | 1 | 3 |
| Surendran, Alsulami, et al., 2019 | 1 | 1 | 1 | 1 | 1 | 1 | -1 | 1 | 6 |
| Surendran, Jayashri, et al., 2019 | 1 | 1 | 1 | 1 | 1 | 1 | -1 | 1 | 6 |
| Alathari et al., 2020 | 1 | 1 | 0 | 1 | 1 | 1 | -1 | 1 | 5 |
| Alsulami et al., 2021 | 1 | 1 | 0 | 1 | 1 | 1 | 0 | 1 | 6 |
| Vimaleswaran et al., 2021 | 1 | 1 | 1 | 1 | 1 | 1 | -1 | 1 | 6 |
| Rana, Sultana, & Bhatti, 2021 | 1 | 1 | 1 | 1 | 1 | 1 | 0 | 1 | 7 |
| Wuni et al., 2022 | 1 | 1 | 0 | 1 | 1 | 1 | -1 | 1 | 5 |

**Table S 5:** Summary Outcome of Assessment with the Appraisal Tool for Cross-Sectional Studies (AXIS)

| Study | 1 | 2 | 3 | 4 | 5 | 6 | 7 | 8 | 9 | 10 | 11 | 12 | 13 | 14 | 15 | 16 | 17 | 18 | 19 | 20 |
| --- | --- | --- | --- | --- | --- | --- | --- | --- | --- | --- | --- | --- | --- | --- | --- | --- | --- | --- | --- | --- |
| Corella et al., 2006 | Y | Y | Y | Y | Y | Y | N | Y | Y | Y | Y | Y | N | Y | Y | Y | Y | Y | N | Y |
|  |  | | | | | | | | | | | | | | | | | | | |
| Corella et al., 2011 | Y | Y | Y | Y | Y | Y | N | Y | Y | Y | Y | Y | N | N | Y | Y | Y | Y | N | Y |
|  |  | | | | | | | | | | | | | | | | | | | |
| Ahmad et al., 2016 | Y | Y | Y | Y | Y | Y | N | Y | Y | Y | Y | Y | N | Y | Y | Y | Y | Y | N | Y |
|  |  | | | | | | | | | | | | | | | | | | | |
| Bodhini et al., 201) | Y | Y | Y | Y | Y | Y | N | Y | Y | Y | Y | Y | N | Y | Y | Y | Y | Y | N | Y |
|  |  | | | | | | | | | | | | | | | | | | | |
| Merritt, Jamnik, & El-Sohemy, 2018 | Y | Y | Y | Y | N | Y | N | Y | Y | Y | Y | Y | N | Y | Y | Y | Y | Y | Y | Y |
|  | 5. Sample size not generalizable  19. Possible conflicts of interest: One author holds shares in Nutrigenomix Inc., a genetic testing company for personalized nutrition, and two other authors declare that they have no competing interests. | | | | | | | | | | | | | | | | | | | |
| Surendran, Alsulami, et al., 2019 | Y | Y | N | Y | Y | Y | Y | Y | Y | Y | Y | Y | N | Y | Y | Y | Y | Y | N | Y |
|  |  |  |  |  |  |  |  |  |  |  |  |  |  |  |  |  |  |  |  |  |
| Surendran, Jayashri, et al., 2019 | Y | Y | N | Y | Y | Y | N | Y | Y | Y | Y | Y | N | N | Y | Y | Y | Y | N | Y |
|  |  |  |  |  |  |  |  |  |  |  |  |  |  |  |  |  |  |  |  |  |
| Alathari et al., 2020 | Y | Y | N | Y | Y | Y | N | Y | Y | N | Y | Y | N | N | Y | Y | Y | Y | N | Y |
|  |  |  |  |  |  |  |  |  |  |  |  |  |  |  |  |  |  |  |  |  |
| Alsulami et al., 2021 | Y | Y | N | Y | Y | Y | N | Y | Y | Y | Y | Y | N | N | Y | Y | Y | Y | N | Y |
|  |  |  |  |  |  |  |  |  |  |  |  |  |  |  |  |  |  |  |  |  |
| Vimaleswaran et al 2016 | Y | Y | Y | Y | Y | Y | N | N | N | N | N | N | N | N | N | N | N | N | N | N |
|  |  |  |  |  |  |  |  |  |  |  |  |  |  |  |  |  |  |  |  |  |
| Vimaleswaran et al., 2021 | Y | Y | Y | Y | Y | Y | Y | Y | Y | Y | Y | Y | N | N | Y | Y | Y | Y | N | Y |
|  |  |  |  |  |  |  |  |  |  |  |  |  |  |  |  |  |  |  |  |  |
| Wuni et al., 2022 | Y | Y | N | Y | Y | Y | N | Y | Y | Y | Y | Y | N | N | Y | Y | Y | Y | N | Y |
|  |  |  |  |  |  |  |  |  |  |  |  |  |  |  |  |  |  |  |  |  |

**Table S 6:** Summary outcome of assessment using the Risk of Bias in Non-Randomized Studies – of Interventions (ROBINS-I)

|  | Bias due confounding | Bias in selection of participants | Bias in classification of exposure | Bias due deviations from intended exposure | Bias due missing data | Bias in measurement of outcome | Bias in selection of reported result | Overall judgment |
| --- | --- | --- | --- | --- | --- | --- | --- | --- |
| Ahmad et al., 2015 | Low | Moderate | Serious | Low | Moderate | Moderate | Low | serious |
| Uma Jyothi & Reddy, 2015 | Low | Moderate | Moderate | Low | Low | Moderate | Low | Moderate |
| Rana, Sultana, & Bhatti, 2021 | Low | Moderate | Moderate | Low | Low | Moderate | Low | Moderate |

**
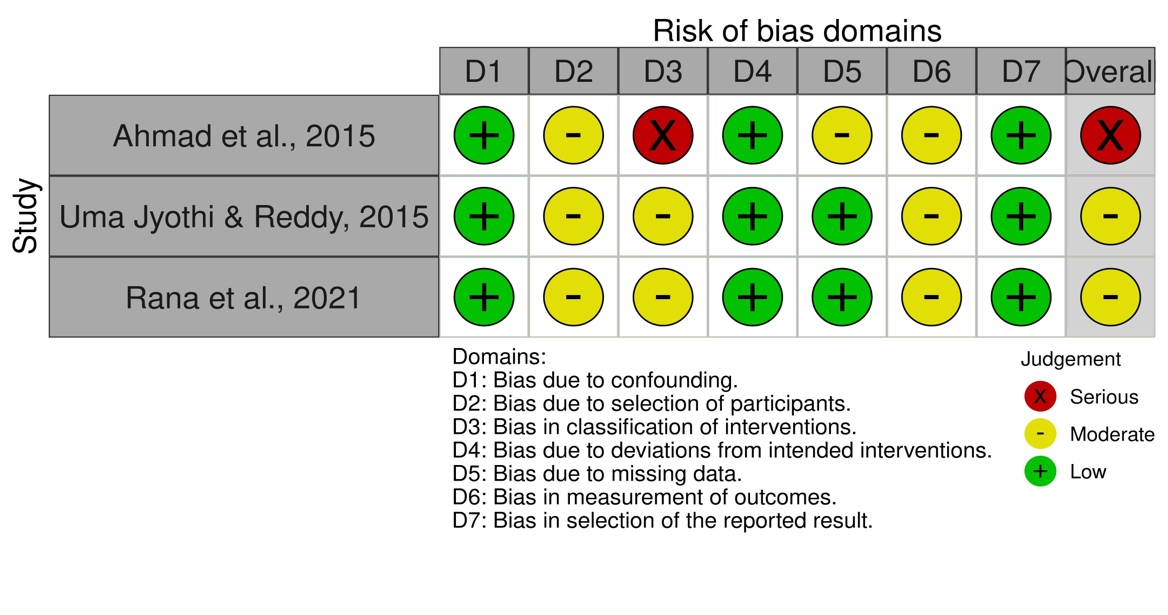
**

**Figure S 1:** Risk of bias assessment of articles included in the narrative synthesis. Overall score: the risk of bias was judged as low risk (green), some concerns (yellow), and high risk (red).
